# Resilience of Epiphytic Lichens to Combined Effects of Increasing Nitrogen and Solar Radiation

**DOI:** 10.3390/jof7050333

**Published:** 2021-04-26

**Authors:** Lourdes Morillas, Javier Roales, Cristina Cruz, Silvana Munzi

**Affiliations:** 1 Centre for Ecology, Evolution and Environmental Changes, Faculdade de Ciências, Universidade de Lisboa, Campo Grande, Bloco C2, 1749-016 Lisbon, Portugal; jroabat@upo.es (J.R.); cmhoughton@fc.ul.pt (C.C.); ssmunzi@fc.ul.pt (S.M.); 2 Departamento de Sistemas Físicos, Químicos y Naturales, Universidad Pablo de Olavide, Ctra. Utrera Km 1, 41013 Seville, Spain; 3 Centro Interuniversitário de História das Ciências e da Tecnologia Faculdade de Ciências, Universidade de Lisboa, Campo Grande, 1749-016 Lisbon, Portugal

**Keywords:** nitrogen pollution, forest decay, global change, chlorophyll fluorescence, Fv/Fm ratio, mediterranean ecosystems

## Abstract

Lichens are classified into different functional groups depending on their ecological and physiological response to a given environmental stressor. However, knowledge on lichen response to the synergistic effect of multiple environmental factors is extremely scarce, although vital to get a comprehensive understanding of the effects of global change. We exposed six lichen species belonging to different functional groups to the combined effects of two nitrogen (N) doses and direct sunlight involving both high temperatures and ultraviolet (UV) radiation for 58 days. Irrespective of their functional group, all species showed a homogenous response to N with cumulative, detrimental effects and an inability to recover following sunlight, UV exposure. Moreover, solar radiation made a tolerant species more prone to N pollution’s effects. Our results draw attention to the combined effects of global change and other environmental drivers on canopy defoliation and tree death, with consequences for the protection of ecosystems.

## 1. Introduction

In the last decade, there has been a growing awareness of forests worldwide being affected by severe decline and mortality driven by biotic and abiotic factors, probably a consequence of global change [1,2,3]. Particularly, in Mediterranean ecosystems located in southern Europe, a severe decline of *Quercus suber* and *Quercus ilex* has been detected since the early 1980s [4,5,6,7]. In a context where human-induced stressors (such as global warming, air pollutants, and invasive exotic species, among others) are weakening and predisposing trees to defoliation and death, there is a pressing need to understand how these changes can translate into disturbed ecosystem functioning [8]. Not only that but also how these changes can interact with other environmental stressors at local or intermediate scales, such as atmospheric pollutants. Nitrogen (N) deposition is one of the most challenging drivers of global change in the study area, as N inputs in the Mediterranean Basin are expected to increase from 7 kg N ha^−1^ yr^−1^ of the mid-1990s to 12 kg N ha^−1^ yr^−1^ in 2050 [9]. Interactions between environmental changes could trigger complex responses subjected to important uncertainties [10,11], which could be pivotal drivers of the ecosystem dynamics [12]. However, our understanding of the consequences of forest decline and its interactions with N deposition is constrained by the limited available tools for disentangling these ecological interactions.

Epiphytic lichens can have a large impact on forest dynamics (e.g., N, carbon (C), phosphorous (P), and water cycling) [13], and due to their ability to regulate canopy environments, can improve water use by plants [14]. Hence, changes in lichen communities could deeply affect other forest processes. To fully understand the implications of the interactive effect of forest decline and increased N inputs for ecosystem functioning, it becomes fundamental to assess their impacts on epiphytic lichens.

A direct consequence of tree defoliation and mortality is the substantial increase in light intensity and, therefore, temperature for epiphytic lichens inhabiting the bark and branches. Such alterations can have a strong effect on lichens, which are amongst the most sensitive organisms to environmental changes [15], providing meaningful ecological systems to model and foresee the response of other less sensitive organisms of the ecosystem [16,17]. These poikilohydric organisms are especially sensitive to climate [15,18] and air pollution [19,20,21] because their physiology is tightly linked to their water status, and they lack mechanisms to control their water and nutrient contents [22]. For this reason, they have been considered as valuable ecological indicators of environmental factors such as temperature and N deposition [15,21,23].

Lichen functional groups are defined as groups of species that respond in a similar way to specific environmental factors [24]. To attribute a species to a given functional group, researchers assess the data available in the literature (e.g., LIAS [25]; Nimis and Martellos [26]; United States Forest Service [27]), and this knowledge has frequently been used to support environmental policies [28]. Therefore, the scientific community has considered of pivotal relevance to keep knowledge about species used in monitoring surveys as detailed and updated as possible [21]. This has been the case for lichens used to identify N pollution status: Oligotrophic species are highly sensitive to N pollution, whereas nitrophytic species tolerate such conditions [16]. This way, for example, it is currently well documented that *Evernia prunastri* (L.) and species belonging to the genus *Usnea* are sensitive species that tend to be affected by increased N availability [29,30,31,32], while *Xanthoria parietina* (L.) is much more resilient to this pollutant [16,30,31]. Despite the great efforts to increase our knowledge on the lichen community response to specific environmental factors, there is great uncertainty on how lichens will respond to the synergistic effect of multiple environmental changes. In particular, the combined effect of exposure to solar irradiation and increasing N availability on lichens has never been studied. Similarly, there is very little information about the recovering capacity of lichens after exposure to stress and none about the above-mentioned factors. Filling these gaps is vital to get a comprehensive understanding of the potential effects of global change, not only on lichen communities but also on important ecosystem processes, which is required for the science-based establishment of environmental policies.

The aim of this research was to assess the combined effect of both increased solar exposure and increased N deposition on the physiological response of six epiphytic lichen species belonging to various functional groups in terms of solar radiation and N tolerance. We hypothesized that the physiological response of lichens to increased solar radiation would be modulated by the N addition treatment, meaning a lower vitality in N-treated samples exposed to direct sunlight than in control ones. We also expected that the different species would respond differently to these environmental factors because of their adaptation strategies. Namely, we expected that the most tolerant species to each one of these environmental stressors would be the most resilient to their synergistic effect. Finally, we hypothesized that species belonging to more tolerant functional groups would show a higher capacity to recover after the stress exposure than sensitive species. To test our hypotheses, we used chlorophyll *a* fluorescence as a sensitive but non-destructive method for assessing the response of photosynthetic organisms to environmental changes [33]. Understanding the response of these sensitive elements of ecosystems to multiple environmental stressors is a primary goal to improve the use of lichens as indicators for environmental protection and predict the potential consequences of global change on ecosystem functioning.

## 2. Materials and Methods

### 2.1. Lichen Sampling

In spring 2020, samples of *Xanthoria parietina*, *Ramalina lacera*, *Usnea* sp., *Flavoparmelia caperata*, *Parmotrema hypoleucinum*, and *Evernia prunastri* were collected from a forest patch near Vila Franca de Xira, Portugal (191 m a.s.L., 38°58′29″ N, 9°00′27″ W). This site was devoid of important sources of pollution; however, it can receive low N deposition from local roads and agricultural areas. Whenever possible, lichens were collected whole by cutting the branch or removing the bark that acted as a substrate. Samples were then transported to the laboratory, where branches or bark were carefully cut in order to leave only the minimum necessary to support the lichen. Finally, lichens were carefully cleaned to remove any impurities. All lichens were stored at room temperature and were fully rehydrated before performing any measurements.

The Ecological Indicator Values (Table 1) established by Nimis and Martellos [26] were used in this study as a proxy of functional groups. This table shows categorical values ranging from 1 to 5 that represent the ecological situations regarding solar irradiation and eutrophication where these species can be found in nature. Although samples of *Usnea* were not identified to species, most species of this genus have very similar ecological requirements with high tolerance to solar radiation and high sensitivity to N.

### 2.2. Nitrogen Treatment

Two levels of N treatment (N 25 and N 50) and a control were established. Treatments involved daily immersion of the samples in 25 mM (N 25) or 50 mM (N 50) (NH_4_)_2_SO_4_ solutions for 5 min repeated for 58 days. The same protocol was followed for control samples using only mineral water with low mineral content. It is worth noting that all species in this experiment have a trebouxioid photobiont, which requires alternate wetting and drying to get a photosynthetic response of the photobiont. Lichens were housed in custom-built wire mesh cages that prevented their flotation on the solutions (see Figure S1 in the Supplementary Materials). After each treatment, the samples were placed in wire mesh shelves in a well-aerated location, which ensured enough drying before the subsequent immersion and hence prevented rotting of the lichen thalli or the substrate (see Figure S2 in the Supplementary Materials). Mineral water with low mineral content was used to avoid osmotic shock. For each treatment, 5 replicates were used.

### 2.3. Increased Radiation Treatment

Light exposure treatment was undertaken in 3 stages in parallel with the N treatment. First, lichens were incubated indoors in front of a big north-facing window, with abundant light but no direct sun. This started the same day as the N treatment and lasted 20 days. The 2nd stage involved the daily exposure of the lichens to 5 h of direct sunlight during the hours of higher solar radiation (from 10 a.m. to 3 p.m., solar time). The average temperature during the exposure was 28 °C, and the maximum temperature was 35.8 °C, with sunny conditions throughout the exposure. The reported temperature was measured in the closest meteorological station, therefore, in shaded conditions. It should be noted that the temperature at the lichen surface, which was permanently facing the sun, was likely to exceed the measured air temperature considerably. This part of the treatment lasted 8 days and was performed in mid-May in southern Spain (42 m a.s.l., 37°16′51″ N, 5°54′54″ W). After each sun exposure, lichens were returned indoors to avoid humidity or temperature fluctuations. In the last stage, intended as recovery, lichens were returned to the indoors location. The recovery lasted 29 days until the end of the experiment. No control group was established for this treatment because we intended to analyze the difference among N levels and not radiation levels.

### 2.4. Chlorophyll a Fluorescence

Treatment effects were quantified using a Plant Efficiency Analyzer Handy PEA (Hansatech Instruments LTD, Pentney, UK), which determined the Fv/Fm ratio, the most frequently used chlorophyll *a* fluorescence parameter, vitality index in ecological research [34,35,36]. Ten minutes after each daily N treatment, with the samples fully rehydrated, the Fv/Fm ratio was measured [37,38]. Lichens were dark-adapted at room temperature for 15 min to maximize oxidation of the primary quinone electron acceptor of PSII immediately before measuring fluorescence. For each treatment, 5 replicates were used.

### 2.5. Statistical Analyses

Data were checked for conformity with repeated measures ANOVA assumptions via Shapiro–Wilk normality test and Mauchly’s test of sphericity. Normality tests of the residuals by time point revealed that they followed approximately a normal distribution. Sphericity could not be assured due to the number of samples being lower than the number of repeated measures, hence a Greenhouse–Geisser correction was used. Differences in Fv/Fm values among N treated and control samples were evaluated via repeated measures procedure following a 2-way mixed ANOVA design with 1 within-subjects factor and 1 between-groups factor in IBM SPSS Statistics 23.0 (SPSS Inc., Chicago, IL, USA). Pairwise comparisons were performed by comparing main effects through post hoc tests using the Bonferroni correction. To investigate interactions, data were divided into subsets based on N treatments and then were subjected to repeated-measures analyses. The evolution following the recovery period was computed as the difference between Fv/Fm values at day 58 (final time) and Fv/Fm values at day 29 (end of the second stage of the radiation treatment). To test for differences in the ability to recover from the radiation treatment of the different N treatments, we performed 1-way ANOVA of the differences between Fv/Fm values for all the studied epiphytic lichens.

## 3. Results

### 3.1. Analyses of the Effect of N Pollution

**Figure 1:**
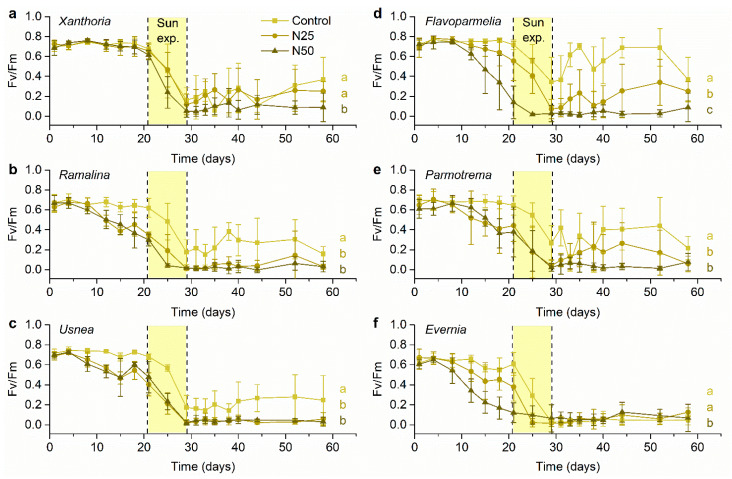
Temporal evolution of Fv/Fm ratio during 58 days for Control (no nitrogen [N] addition), 25 N (treated with 25 mM of (NH_4_)_2_SO_4_) and 50 N (treated with 50 mM of (NH_4_)_2_SO_4_) for the following species: (**a**) *Xanthoria parietina*, (**b**) *Ramalina lacera*, (**c**) *Usnea* sp., (**d**) *Flavoparmelia caperata*, (**e**) *Parmotrema hypoleucinum*, (**f**) *Evernia prunastri*. All samples were subjected to the same radiation treatment. The period of exposure to direct sunlight is indicated in yellow. Lowercase letters indicate significant differences among N treatments for each species (*n* = 5).

Six measurements over the first 20 days assessed the effects of N pollution alone, while lichens were not subject to irradiation. Significant effects of the different N treatments and time for all species except *Xanthoria* were found (Figure 1 and Table 2). For all affected species, Fv/Fm values gradually dropped, responding to the cumulative effect of N, especially the 50 N dose (Figure 1). In *Evernia* and *Flavoparmelia* there was no significant effect of the low N dose but a significant reduction with the high N dose compared with both the control and low N dose. In *Parmotrema*, *Ramalina* and *Usnea,* both N doses caused significant reductions, but the level of reduction between the N doses was statistically indistinguishable. Measurements of the control indicated there was no deterioration in the index due to time, i.e., the experimental conditions did not affect the measurements. However, with the N treatments, as the experimental duration progressed, the lichens received more N, i.e., time also means a greater cumulative N dose. We detected significant differences between the control and 50 N and 25 N and 50 N but not between control and 25 N for *Evernia* (*p*_control-25 N_ = 0.960, *p*_control-50 N_ < 0.0005, *p*_25 N–50 N_ < 0.0005) and *Flavoparmelia* (*p*_control-25 N_ = 0.534, *p*_control-50 N_ = 0.001, *p*_25 N–50 N_ = 0.009). These differences were also found among control and N-treated samples, both 25 and 50 N, although not between them, for *Parmotrema* (*p*_control-25 N_ = 0.002, *p*_control-50 N_ = 0.002, *p*_25 N–50 N_ = 1.000), *Ramalina* (*p*_control-25 N_ < 0.0005, *p*_control-50 N_ < 0.0001, *p*_25 N–50 N_ = 1.000) and *Usnea* (*p*_control-25 N_ < 0.0005, *p*_control-50 N_ < 0.0005, *p*_25 N–50 N_ = 1.000). There was a significant N treatment × time interaction for *Ramalina*, *Flavoparmelia*, *Usnea*, and *Evernia*, and when looking into them, we found a significant effect of time for 25 N and 50 N treatments, but not for control for *Ramalina*, *Usnea*, and *Evernia* (Table 2). In the case of *Flavoparmelia*, we only detected a significant effect of time for 50 N (Table 2).

### 3.2. Analyses of the Synergetic Effect of N and Solar Radiation

We found a significant effect of the different N treatments on all lichen species when analyzing the synergetic effect of N and increased solar radiation (Figure 1 and Table 3). For all species, direct sun exposure dramatically decreased Fv/Fm (Figure 1), although these values reached their minimum earliest in N treated samples. Lichens exposed to 50 N treatment showed a consistent trend towards lower Fv/Fm values for all species (Figure 1). We detected significant differences between the control and 50 N and 25 N and 50 N but not between control and 25 N for *Xanthoria* (*p*_control-25 N_ = 1.000, *p*_control-50 N_ = 0.003, *p*_25 N–50 N_ = 0.009) and *Evernia* (*p*_control-25 N_ = 0.133, *p*_control-50 N_ < 0.0005, *p*_25 N-50 N_ = 0.009). Differences were also found among control and N-treated samples, both 25 and 50 N, although not between them, for *Parmotrema* (*p*_control-25 N_ < 0.0005, *p*_control-50 N_ < 0.0005, *p*_25 N–50 N_ = 0.072), *Ramalina* (*p*_control-25 N_ < 0.0005, *p*_control-50 N_ < 0.0005, *p*_25 N–50 N_ = 0.282) and *Usnea* (*p*_control-25 N_ < 0.0005, *p*_control-50 N_ < 0.0005, *p*_25 N–50 N_ = 1.000). We found significant differences among all the three groups (control, 25 N and 50 N) for *Flavoparmelia* (*p*_control-25 N_ < 0.0005, *p*_control-50 N_ < 0.0005, *p*_25 N–50 N_ < 0.0005). Time was found to significantly affect all analyzed lichens species (Figure 1 and Table 3). There was a significant N treatment × time interaction for *Ramalina*, *Flavoparmelia,* and *Evernia*, and when looking into them, we found a significant effect of time for the control, 25 N and 50 N treatments for all these species (Table 3).

### 3.3. Analyses of the Evolution Following the Recovery Period

**Figure 2:**
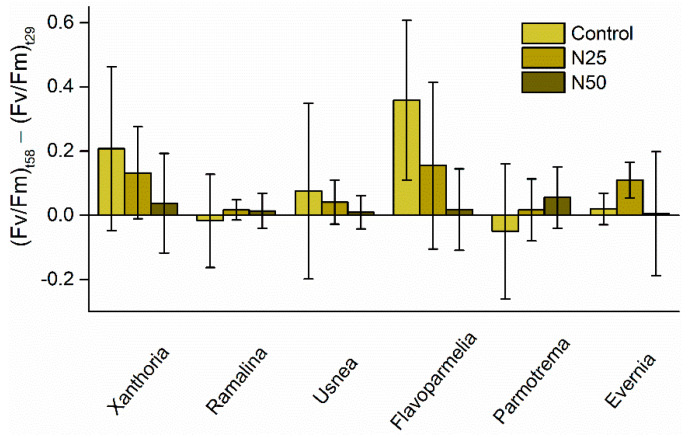
Recovery capacity for all studied lichens computed as the difference between Fv/Fm ratio at the end of the experiment (day 58) and Fv/Fm ratio at the end of the direct sunlight exposure (day 29) for the control (no nitrogen [N] addition), 25 N (treated with 25 mM of (NH_4_)_2_SO_4_), and 50 N (treated with 50 mM of (NH_4_)_2_SO_4_). No significant differences among N treatments were found for any species (*n* = 5).

None of the studied species was able to recover following the solar radiation exposure (Figure 1 and Figure 2). However, a non-significant trend of decreased ability to recover from solar radiation stress with increased N dose was found for *Xanthoria*, *Usnea*, and *Flavoparmelia* (Figure 2 and Table 4).

## 4. Discussion

In general, our results showed a detrimental cumulative effect of N and solar radiation exposure on lichens but did not support the view that increased N availability constrained the physiological ability of lichens to cope with increased sunlight exposure, disproving our first hypothesis. However, in the case of *Xanthoria*, we found a differential response: It was unaffected by N addition, but solar exposure promoted an N dose-dependent response of the Fv/Fm parameter. Therefore, the physiological response of this species to increased N addition was modulated by solar radiation. Our second hypothesis, which states that different lichen species would vary in their response to the synergetic effect of increased N doses and solar exposure, was not supported by our data; we expected that the most tolerant species to each one of these environmental stressors, i.e., *Xanthoria*, would be the most resilient to their synergistic effect. In contrast to our expectations, we only found slight differences among species, as at the end of the combined treatment, *Xanthoria* was affected to the same extent as the rest of the analyzed species. Similarly, our last hypothesis was not supported because more tolerant functional groups were equally unable to recover after sunlight exposure as sensitive species.

These results indicate that a reduction of vitality among the epiphytic community should be expected in the face of global change. Increased N deposition that is projected to occur in the next decades in Mediterranean regions [9] has previously been pinpointed as a key factor underpinning reduced vitality in many epiphytic lichens [39]. Our data during 20 days of N addition, pre solar radiation, support this view, with reduced vitality in N sensitive species. Other researchers have found a fertilizing effect of moderate N supply shown by increased thalli N, chlorophyll concentration, and rates of photosynthesis [21,40]. It is well documented that N performs as a nutrient below the toxicity threshold, especially in oligotrophic environments [41,42,43]. However, we did not observe this, possibly because the lichens came from a site with limited N contribution from surrounding roads and agricultural areas. Thus, even modest N addition doses during our experiment exceeded the fertilization threshold and performed as a pollutant.

Direct exposure to high solar radiation can also cause stress and threaten epiphytic lichens. Some lichens appear to be highly sensitive to sudden solar exposure increase [44], such as that observed in anthropogenically induced forest decline. This is despite the varied protective mechanisms lichens employ to avoid damage from high insolation such as colored cortical secondary compounds (e.g., usnic acid, parietin, and melanic compounds, [45,46]), thick cortical layer [47,48,49], hairiness [50], light-reflecting calcium oxalate crystals, or biochemical protective mechanisms [51]. A number of studies report a high natural radiation susceptibility of photosystem II in lichens [44,52], with particular sensitivity to both excessive ultraviolet (UV) [53] and photosynthetically active radiation [54]. Likewise, the increased temperature has also been highlighted as a major damaging factor for epiphytic lichen vitality. Smith and collaborators [55] found that warm climate tolerant lichen communities are already close to exceeding their upper climatic limits and are even more vulnerable to increased temperatures than high-elevation lichens. Our experimental design, which accounted for exposing wet lichens to direct solar radiation, does not allow us to discriminate between the effect of increased radiation and temperature. However, when Gauslaa and Solhaug [56] enclosed thalli of various lichens in a desiccator at 15 °C illuminated at 100 mol m^−2^ s^−1^, periodic measurements of Fv/Fm showed a decreasing trend. They then removed all uppermost thalli exposed to the irradiance and measured thalli that had been shaded by the previously harvested thalli, finding that Fv/Fm values suddenly rose close to pre-desiccation levels (Gauslaa and Solhaug, unpublished data). Based on these findings, it is reasonable to think that the damaging factor operating in our experiment could be increased radiation more than temperature, although the interactive effect of both factors is also a plausible possibility. Accordingly, Gauslaa and Solhaug [57] reported a photoinhibition effect on lichen thalli due to heat and light stress both separately and combined.

Hydration status is a key determinant in how lichens respond to radiation. Wet thalli are more susceptible to irradiance than dried thalli because humidity increases the translucency of the protective upper cortex, increasing light absorbance [58,59]. Therefore, photosystem II needs more protection against the harmful excess light energy compared to desiccated thalli [60], in which case most of the radiation is reflected from the thalli surface [58]. Besides this generic aspect that might have had a role in the striking and undivided negative response of all studied lichens to solar radiation, hydration appears to deeply affect photoinhibition in a species-specific manner. Looking at the control thalli, the least affected by solar radiation was *F. caperata*. This could appear in contradiction with the fact that *F. caperata* is the only one among the species used in the experiment, adapted to diffuse light more than to direct solar radiation [26]. However, shade-adapted species were found to be most affected by radiation when they are dry, whereas sun-adapted species tend to be more susceptible when they are wet [56]. Tretiach et al. [61] showed that when hydrated, *F. caperata* activate repair mechanisms like ROS-scavenging enzymes and oxidation of polyols and phenols that allow it to survive in case of environmental stress. Since our samples were fully hydrated every day, we can reasonably argue that thalli could restore a proper enzymatic activity and antioxidants concentration daily [62]. In agreement, Gauslaa and Solhaug [56] observed that the sun-adapted *X. parietina* was less affected by radiation in its desiccated state, whereas hydration caused increased photoinhibition. These authors concluded that the extent of solar damage varies more among species when dry lichens are exposed to radiation than when radiation affects wet lichens. This, and the fact that all tested lichens shared a trebouxioid photobiont fully active following alternate wetting and drying cycles, could explain why we found such a homogeneous response to direct insolation among all studied species. Even more, this differential species-specific response to direct radiation responding to the hydration status could be the reason why we did not find the expected tolerance to radiation in *Xanthoria* when compared to shade-adapted species, as reported by other authors [26]. In other words, hydration status might be modulating the lichens species-specific response to excess irradiance.

The synergetic effect of N pollution and increased solar exposure (Table 2) did not differentiate between the lichens, but there were species response differences to N alone (Table 3). As expected, *Xanthoria* was the only species avoiding N-related physiological damage. It has been well established that *X. parietina* tolerates high atmospheric concentrations of ammonium in the field [16] and was physiologically unaffected when subjected to NO_3_^−^ and NH_4_^+^ excess in laboratory experiments [30,31]. Likewise, treatment with ammonium nitrate 50 mM for 6 weeks did not cause any significant decrease in the Fv/Fm parameter [39]. However, N does appear to have impaired the photosynthetic response to the secondary stress (Table 2). The highest N dose induced a decrease of the Fv/Fm ratio in *Xanthoria* responding to increased solar radiation when compared to the control and the lowest N dose (Figure 1). These results suggest that costly mechanisms to overcome harmful effects of increased solar irradiance might have compromised the lichen ability to make the needed investment in N tolerance. Nitrogen tolerance in lichens seems to be provided by constitutive characteristics [63,64], but also by other inducible metabolic mechanisms [43,65,66]. The activation of such protective mechanisms comes at a cost, as shown by proteomic analysis in thalli of *Cladonia portentosa*: In thalli exposed to long-term N treatments, the ability to cope with increased N availability was related to an enhanced energetic metabolism [67]. In contrast, UV protection and tolerance to high solar radiation are provided by specific sunscreen compounds (e.g., usnic acid, phenolics, parietin [68,69,70]) and a general capacity to avoid and repair damages of oxidative stress [71]. As for N protection strategies, both the synthesis of secondary metabolites and antioxidant activities require energy [69]. Thus, *Xanthoria* could have spent more resources to respond to solar radiation, like the production of secondary compounds in the mycobiont, such as parietin, that can perform as regulators to avoid damage from high solar radiation [45,46], reducing other tasks. Nevertheless, we cannot unequivocally attribute this process to our results because measurements of secondary compounds would be needed to test this hypothesis.

As for the recovery capacity, in the light of the results shown in Figure 2 together with the bibliographic review, we deem the main part of the measured reduction in Fv/Fm values to be long-lasting photoinhibitory radiation damage. According to Solhaug and Gauslaa [72], a 2-day recovery period must be used to identify the more permanent long-term photoinhibitory damage to photosystem II in *X. parietina*, thus the 29-days recovery period used in our experiment is more than enough to classify this damage as permanent long-term. Such large depressions relative to start values responding to solar radiation in all species along with the lack of difference between species and N treatments in the recovery capacity, indicated that neither their functional groups nor the N treatments have a role in modulating the detrimental effect of solar radiation on epiphytic lichens. Gauslaa and Solhaug [56] observed that in some lichens, photosystem II is highly susceptible to solar radiation, preventing them from complete recovery even after extended recovery periods at low light that, according to Ögren [54], should allow recovery from photoinhibition.

## 5. Conclusions

Our results demonstrate the highly relevant consequences of the synergistic effects of N pollution and increased solar radiation for epiphytic lichens, likely moderated by the hydration status. Besides direct pollutant effects, the global change-driven forest decline promoting tree defoliation and death is likely to exacerbate the impact of N deposition on epiphytic lichens, making lichen species considered tolerant more prone to N pollution effects. Furthermore, the high susceptibility of photosystem II in epiphytic lichens should be taken into account in future studies on the synergetic effect of this environmental factor along with other global change drivers potentially inducing lichen damage. The loss of these organisms would likely have consequences for forest functions such as water retention, cycling of N, C, and P, as well as the provision of wildlife habitat [13]. Our results draw attention to the need to protect ecosystems from environmental drivers threatening canopy defoliation and tree death.

## Figures and Tables

**Table 1:** Lichen’s Ecological Indicator Values established by Nimis and Martellos (2020).

Lichen Species	Solar Irradiation ^1^	Eutrophication ^2^
*Xanthoria parietina*	3, 4, 5	3, 4
*Ramalina lacera*	4, 5	2, 3
*Usnea* sp.	4, 5	1, 2
*Flavoparmelia caperata*	3, 4	1, 2, 3
*Parmotrema hypoleucinum*	4, 5	1, 2
*Evernia prunastri*	3, 4, 5	1, 2, 3

^1^ Values mean: 1 in very shaded situations, 2 in shaded situations, 3 in sites with plenty of diffuse light but scarce direct solar irradiation, 4 in sun-exposed sites, 5 in sites with very high direct solar irradiation. ^2^ Values mean: 1 no eutrophication, 2 very weak eutrophication, 3 weak eutrophication, 4 rather high eutrophication, 5 very high eutrophication.

**Table 2:** Repeated measures ANOVA analyses for all the studied epiphytic lichens during the first 20 days. The samples were subjected to N treatment alone during this period. Control = No nitrogen (N) addition, 25 N = treated with 25 mM of (NH_4_)_2_SO_4_, 50 N = treated with 50 mM of (NH_4_)_2_SO_4_.

Lichen Species	Factor	df	F	*p*
*Xanthoria parietina*	N treatment	2	0.198	0.823
	Time	2.870	1.912	0.148
	N treatment × Time	5.740	0.617	0.709
*Ramalina lacera*	N treatment	2	23.679	<0.0005
	Time	2.707	20.028	<0.0005
	N treatment × Time	5.413	3.857	0.006
Interaction N treatment × Time	Control	1.999	0.920	0.437
Interaction N treatment × Time	25 N	1.369	17.619	0.005
Interaction N treatment × Time	50 N	2.247	9.150	0.006
*Usnea* sp.	N treatment	2	22.354	<0.0005
	Time	2.418	19.147	<0.0005
	N treatment × Time	4.836	3.428	0.016
Interaction N treatment × Time	Control	2.102	3.194	0.091
Interaction N treatment × Time	25 N	2.752	15.265	<0.0005
Interaction N treatment × Time	50 N	1.458	6.499	0.038
*Flavoparmelia caperata*	N treatment	2	14.098	0.001
	Time	2.310	7.787	0.001
	N treatment × Time	4.620	4.170	0.007
Interaction N treatment × Time	Control	2.612	1.626	0.243
Interaction N treatment × Time	25 N	2.133	3.792	0.064
Interaction N treatment × Time	50 N	2.060	5.772	0.027
*Parmotrema hypoleucinum*	N treatment	2	14.291	0.001
	Time	3.032	5.077	0.005
	N treatment × Time	6.064	1.531	0.195
*Evernia prunastri*	N treatment	2	34.371	<0.0005
	Time	3.066	28.366	<0.0005
	N treatment × Time	6.132	5.988	<0.0005
Interaction N treatment × Time	Control	1.911	3.591	0.081
Interaction N treatment × Time	25 N	2.283	6.503	0.024
Interaction N treatment × Time	50 N	1.877	21.993	0.001

**Table 3:** Repeated measures ANOVA analyses for all the studied epiphytic lichens during the whole experiment. The samples were subjected to N treatment and solar radiation. Control = No nitrogen (N) addition, 25 N = treated with 25 mM of (NH_4_)_2_SO_4_, 50 N = treated with 50 mM of (NH_4_)_2_SO_4_.

Lichen Species	Factor	df	F	*p*
*Xanthoria parietina*	N treatment	2	10.769	0.002
	Time	4.753	71.389	<0.0005
	N treatment × Time	9.505	1.230	0.293
*Ramalina lacera*	N treatment	2	112.702	<0.0005
	Time	5.020	85.423	<0.0005
	N treatment × Time	10.039	2.083	0.040
Interaction N treatment × Time	Control	3.381	12.826	<0.0005
Interaction N treatment × Time	25 N	2.000	41.841	<0.0005
Interaction N treatment × Time	50 N	2.786	80.237	<0.0005
*Usnea* sp.	N treatment	2	99.448	<0.0005
	Time	4.560	137.635	<0.0005
	N treatment × Time	9.119	1.527	0.164
*Flavoparmelia caperata*	N treatment	2	372.020	<0.0005
	Time	5.163	36.500	<0.0005
	N treatment × Time	10.326	4.744	<0.0005
Interaction N treatment × Time	Control	2.369	4.875	0.031
Interaction N treatment × Time	25 N	2.545	13.058	0.001
Interaction N treatment × Time	50 N	2.425	37.915	<0.0005
*Parmotrema hypoleucinum*	N treatment	2	46.314	<0.0005
	Time	6.299	32.540	<0.0005
	N treatment × Time	12.598	1.629	0.098
*Evernia prunastri*	N treatment	2	21.052	<0.0005
	Time	6.129	135.430	<0.0005
	N treatment × Time	12.258	7.621	<0.0005
Interaction N treatment × Time	Control	2.517	86.264	<0.0005
Interaction N treatment × Time	25 N	2.663	59.673	<0.0005
Interaction N treatment × Time	50 N	3.231	25.576	<0.0005

**Table 4:** One-way ANOVA of the Fv/Fm evolution following the recovery period for all the studied epiphytic lichens.

Lichen Species	df	F	*p*
*Xanthoria parietina*	2	0.876	0.442
*Ramalina lacera*	2	0.304	0.743
*Usnea* sp.	2	0.280	0.761
*Flavoparmelia caperata*	2	2.379	0.135
*Parmotrema hypoleucinum*	2	0.445	0.651
*Evernia prunastri*	2	0.789	0.477

## Data Availability

The data presented in this study are openly available in FigShare at https://doi.org/10.6084/m9.figshare.14473353.v1 (accessed on 15 March 2021).

## Supplementary Materials

The following are available online at https://www.mdpi.com/article/10.3390/jof7050333/s1, Figure S1: Setup for the nitrogen treatment, Figure S2: Shelves containing the lichens during the experiment.
