# Supplementary material for: Resilience of Epiphytic Lichens to Combined Effects of Increasing Nitrogen and Solar Radiation
Source: J Fungi (Basel). 2021 Apr 26;7(5):333. doi: 10.3390/jof7050333 (PMC8145261; doi:10.3390/jof7050333)
Supplement: Supplementary file 1 [file jof-07-00333-s001.zip › supplementary material.pdf]

Supplementary Material - Resilience of Epiphytic Lichens to Combined Effects of Increasing Nitrogen and Solar Radiation

Lourdes Morillas [1,*], Javier Roales [2,1], Cristina Cruz [1] and Silvana Munzi [1,3]

[1] Centre for Ecology, Evolution and Environmental Changes, Faculdade de Ciências, Universidade de Lisboa, Campo Grande, Bloco C2, 1749-016 Lisbon, Portugal;
[2] Departamento de Sistemas Físicos, Químicos y Naturales, Universidad Pablo de Olavide, Ctra. Utrera Km 1, Seville, Spain;
[3] Centro Interuniversitário de História das Ciências e da Tecnologia Faculdade de Ciências, Universidade de Lisboa, Campo Grande, 1749-016, Lisbon, Portugal
* Correspondence: lmorillas@fc.ul.pt (L.M.)

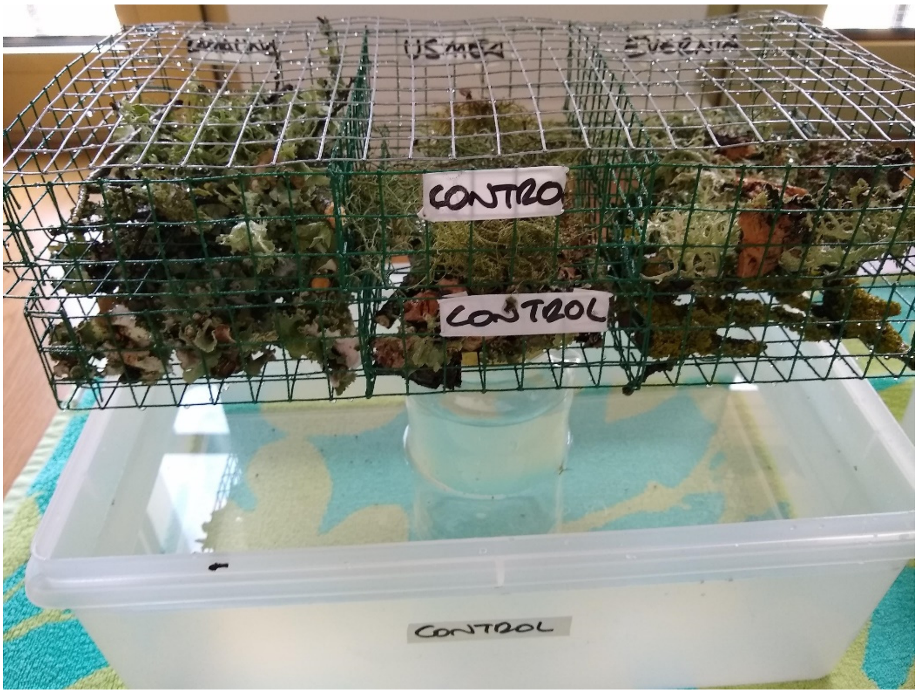

Figure S1. Setup for the nitrogen treatment. Lichens were housed in custom-built wire mesh cages that prevented their flotation on the solutions.

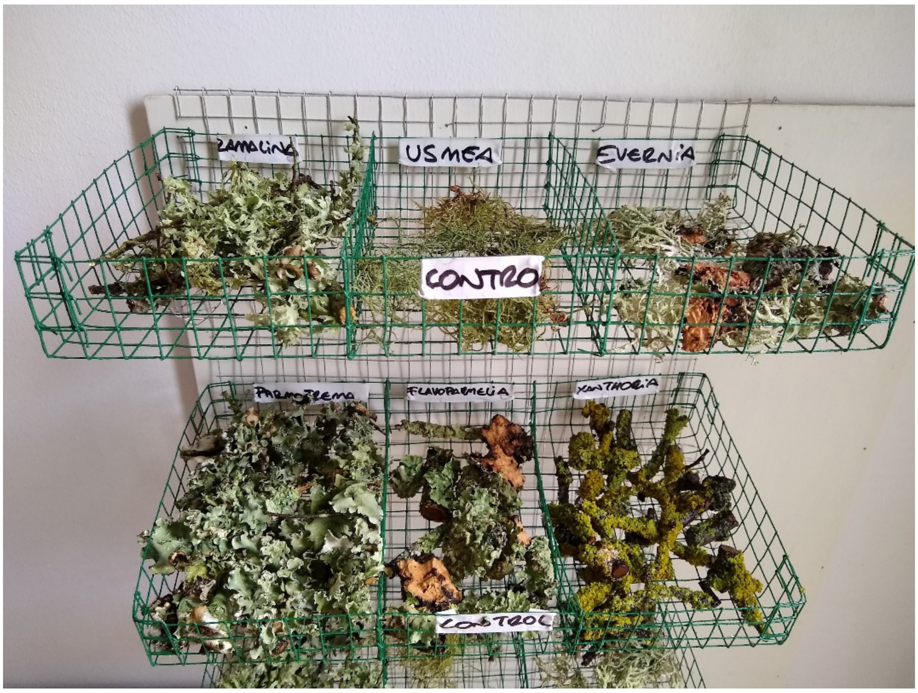

Figure S2. Shelves containing the lichens during the experiment. Lichen samples were placed in wire mesh shelves in a well-aerated location, which ensured enough drying before the subsequent immersion and hence prevented rotting of the lichen thalli or the substrate.

© 2021 by the authors. Submitted for possible open access publication under the terms and conditions of the Creative Commons Attribution (CC BY) license (http://creativecommons.org/licenses/by/4.0/).
